# A Rare Case of Multifocal Papillary Thyroid Cancer in Bilateral Thyroid Cysts

**DOI:** 10.1155/2018/1656831

**Published:** 2018-04-17

**Authors:** Anupama Roy Chowdhury, Jack Kian Ch'ng, Choon Chieh Tan

**Affiliations:** ^1^Department of Internal Medicine, Sengkang General Hospital, Sengkang E Way, Singapore; ^2^Department of Vascular Surgery, Singapore General Hospital, Bukit Merah, Singapore; ^3^Department of General Surgery, Sengkang General Hospital, Sengkang E Way, Singapore

## Abstract

Papillary thyroid cancer (PTC) can present as a thyroid cyst. In its more aggressive form, PTC may be multifocal in nature and is associated with a poorer prognosis. In patients whom PTC is diagnosed incidentally after a diagnostic lobectomy, the decision whether to offer completion thyroidectomy is sometimes challenging to make if such patients fall in the ‘low-to-intermediate' risk category. We present a case of a 55-year-old lady who had a predominantly cystic left thyroid nodule with no suspicious features on ultrasound as well as 2 subcentimetre simple right-sided cysts. She subsequently underwent left hemithyroidectomy, and this reported a T2 PTC in the thyroid cyst. This was followed by completion thyroidectomy which yielded the surprising finding of PTC in the two tiny right sided cysts. This case highlights the need for vigilance in managing patients with thyroid cysts even though thyroid ultrasound scan did not reveal any overt suspicious features.

## 1. Introduction

Multifocality is not uncommon in papillary thyroid cancer (PTC) and is associated with more aggressive tumour behaviour. Cystic thyroid nodules are often benign, and ultrasound surveillance alone is regarded as a reasonable option for cystic nodules with a very low suspicion for malignancy. We report a case of multifocal (MF) cystic PTC that first presented as a solitary thyroid cyst.

## 2. Case Report

**Figure 1:**
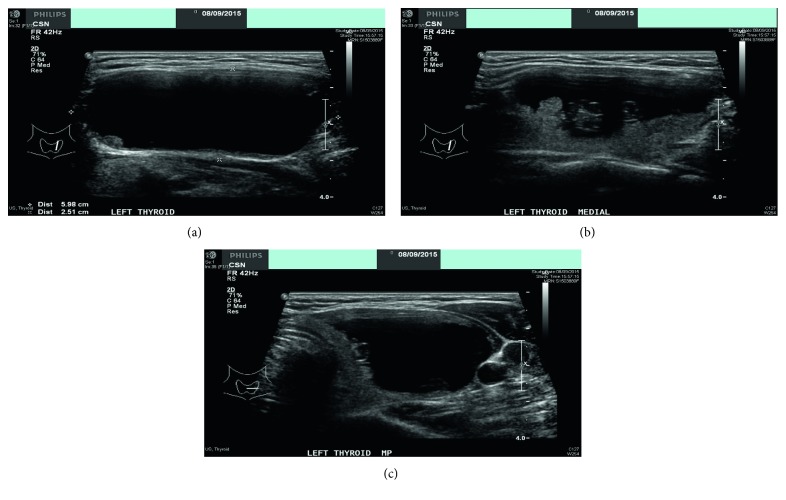
(a) Longitudinal view of the left thyroid cyst, (b) Longitudinal (medial) view of the left thyroid cyst, and (c) Transverse view of the left thyroid cyst.

Mrs C is 55 years of age and presented with a left neck swelling that was noticed incidentally some days prior. She did not have any compressive symptoms related to the neck lump. Clinical examination revealed a 4 cm left smooth thyroid nodule. Her thyroid function test was normal while the ultrasound scan showed a 5.9 by 3.8 cm predominantly cystic nodule with a small solid component (Figure 1). The nodule was smooth and did not demonstrate any increased vascularity. The right lobe had 2 benign looking simple cysts (0.4 by 0.4 cm, 0.3 by 0.3 cm). There was no cervical lymphadenopathy demonstrated.

Ultrasound-guided FNA was performed, and the aspirate reported mucoid fluid suggestive of cyst contents. In view of the size of the left cystic nodule, Mrs C was counselled for a left hemithyroidectomy. This was completed uneventfully, and the final histology was that of a pT2 (2.1 cm) PTC without extrathyroidal extension nor lymphatic invasion. In view of the diagnosis, she opted for a completion right thyroidectomy because she wanted to minimize any risk of disease recurrence and optimize postoperative surveillance. The histology of the right lobe showed 2 foci of PTC each about 3 mm.

Postoperatively, Mrs C underwent a radioiodine scan which did not reveal any distant metastasis. She was also given an ablative dose of RAI to obliterate a small thyroid bed remnant gland and then started on thyroxine replacement. At about 1 yr follow up, the surveillance ultrasound thyroid scan and thyroglobin marker did not show any sign of cancer recurrence.

## 3. Discussion

Multifocal tumours are found in 15.7%–37.2% of PTC patients [1–3], and it has been reported that preoperative prediction of MFPTC is possible in up to 67% of patients [4]. MFPTC is a more aggressive disease compared to unifocal PTC; it is associated with more frequent nodal disease, and the number of tumour foci correlates with LN metastases [2, 5]. Multifocality is also found to be an independent risk factor for neck recurrence, distant metastasis, and cancer death [3, 6]. The incidence of BRAF mutation is also higher in MFPTC compared to unifocal PTC [3]. For the above considerations, total thyroidectomy is often the done for PTCs > 1 cm.

The majority of cystic thyroid nodules are benign and do not require surgical intervention. In their analysis of PTC, Lee et al. found that only 5% of partially cystic lesions were malignant and these nodules were associated with microcalcifications [7]. Likewise, Henrichsen et al. reported that only 2.5 % of carcinoma were predominantly cystic and all were associated with suspicious features such as microcalcifications [8].

Under the 2017 American Thyroid Association guidelines [9], sonographic features suggestive of PTC include the presence of microcalcifications, nodule hypoechogenicity, irregular margins, and a shape taller than wide. Partially cystic nodules without these sonographic features are recommended for FNA only if they are at least 1.5 cm (“low suspicion”), and for nodules stratified under “very low suspicion,” observation without FNA is regarded as a reasonable option. In our case, it was unfortunate that the patient's ultrasound scan did not reveal any worrisome features other than a relatively small eccentric solid component in the left cystic nodule.

Given our patient's left nodule was predominantly cystic in nature, it perhaps comes as no surprise that the FNA could only yield “cyst fluid.” This form of aspirate falls under the “nondiagnostic” category of the Bethesda system, and metaanalysis actually suggests that up to 16.8% of such nodules are malignant [10]. Even the presence of atypical epithelial cells on FNA may not be of additional value because such features are observed in specimens from benign thyroid cysts [11, 12].

In nodules ≥4 cm, ultrasound-guided FNA was found to be less reliable and gave a false negative rate of 10.4% [12]. Because of this limitation, thyroid lobectomy is strongly recommended in nodules that are ≥4 cm. At minimum, repeat FNA ought to be done to exclude a malignancy [13]. This size criterion was the main consideration behind offering surgery to our patient since her FNA was unhelpful, and ultrasound scan stratified her lesion to be at “low suspicion” of malignancy.

Following the diagnosis of PTC in the left thyroid cyst, the question arises on whether the patient requires a completion thyroidectomy. Adopting the 2017 ATA guidelines, “completion thyroidectomy should be offered to patients for whom a bilateral thyroidectomy would have been recommended had the diagnosis been available before the initial surgery.” With a PTC < 4 cm, no evidence of extrathyroidal extension or lymph node metastases by examination or imaging, the patient would fall into the “low-to intermediate-risk” category and in fact qualify for lobectomy only. For patients aged >45 years or have contralateral nodules, a completion thyroidectomy is also an option.

In this case, what the final histology in the right lobe serves as a reminder is that multifocality is a clear and present danger in PTC despite what the preoperative ultrasound imaging revealed, that is, benign features of subcentimetre cysts. Therefore the ATA's consideration of “contralateral thyroid nodules” should be interpreted more liberally such that even benign looking tiny cysts are viewed with caution.

## 4. Conclusion

PTC can present as a cystic thyroid nodule and involve bilateral lobes in its more aggressive form. In the absence of benign cytology on FNA and despite the lack of suspicious features on ultrasonography, cystic thyroid nodules should be treated with caution if they are ≥4 cm, and thus surgery is recommended to avoid missing a thyroid malignancy. Following the diagnosis of PTC, completion thyroidectomy should be offered to a patient if the preoperative ultrasound does not show a pristine contralateral lobe.
